# Effects of long-term supplementation of probiotics on cognitive function and emotion in temporal lobe epilepsy

**DOI:** 10.3389/fneur.2022.948599

**Published:** 2022-07-19

**Authors:** Xue Wang, Rui Ma, Xinyi Liu, Yongbo Zhang

**Affiliations:** ^1^Department of Neurology, Beijing Friendship Hospital, Capital Medical University, Beijing, China; ^2^Department of Neurology, Xuanwu Hospital, Capital Medical University, Beijing, China

**Keywords:** probiotics, cognitive function, temporal lobe epilepsy, supplementation, neuropsychiatric disorders

## Abstract

Cognitive impairment and neuropsychiatric disorders are very common in patients with temporal lobe epilepsy (TLE). These comorbidities complicate the treatment of epilepsy and seriously affect the quality of life. So far, there is still no effective intervention to prevent the development of epilepsy-associated comorbidities. Gut dysbiosis has been recognized to be involved in the pathology of epilepsy development. Modulating gut microbiota by probiotics has shown an antiseizure effect on humans and animals with epilepsy. Whether this treatment strategy has a positive effect on epilepsy-associated comorbidities remains unclear. Therefore, this study aimed to objectively assess the effect of probiotics on cognitive function and neuropsychiatric performance of patients with TLE. Participants enrolled in an epilepsy clinic were randomly assigned to the probiotic and placebo groups. These two groups were treated with probiotics or placebo for 12 weeks, and then the cognitive function and psychological performance of participants were assessed. We enrolled 76 participants in this study, and 70 subjects were finally included in the study (35 in the probiotics group and 35 in the placebo group). Our results showed significant seizure reduction in patients with TLE treated with probiotics. No significant differences were observed on cognitive function (including intelligence and memory) between groups. For neuropsychiatric performances, supplementation of probiotics significantly decreased the Hamilton Anxiety Rating and Depression Scale scores and increased the 89-item Quality of Life in Epilepsy Inventory score in patients with TLE. In conclusion, probiotics have a positive impact on seizures control, and improve anxiety, depression, and quality of life in patients with TLE.

## Introduction

Epilepsy is a very common disease in central nervous system affecting approximately 70 million people worldwide (1). Despite the emergence of multiple antiepileptic drugs (AEDs) for the treatment of epilepsy, approximately one in three patients develop drug-resistant epilepsy (DRE) (2). Temporal lobe epilepsy (TLE) is the most common type of epilepsy, which is prone to develop into DRE. Due to abnormal brain discharges originating focally in the temporal lobe and limbic system involving cognitive function and emotion, patients with TLE often suffer from different degrees of cognitive impairment and neuropsychiatric disorders (3–6). These comorbidities severely affect patients' quality of life and burden the family and society (7). Although various drugs targeting a series of comorbidities have emerged, there is still no effective intervention to prevent comorbidity development in patients with epilepsy. Therefore, understanding the pathophysiology of comorbidities associated with epilepsy may help develop therapeutic interventions.

The gut microbiota has gradually been recognized to play an important role in the pathology of epilepsy development (8–10). The gut-brain axis is a bidirectional communication pathway connecting the central nervous and enteric nervous systems, and modulates the neural, immunological, and hormonal pathways to balance the body (11, 12). Gut dysbiosis changes the levels of neurotransmitters, metabolites, and activities in the gut and affects glial function, neuroinflammation, myelination, blood-brain barrier permeability, and neurotransmission, leading to various neurological disorders (8, 13). Recently, a series of studies showed alterations of the gut microbiome in patients with DRE, such as increased relative abundance of Firmicutes and Proteobacteria and decreased Bacteroidetes and Actinobacteria, suggesting that gut dysbiosis may be involved in the pathogenesis of epilepsy (14, 15). Modulation of gut microbiota may be a potential therapeutic strategy for epilepsy. Another study provided evidence that supplementation with probiotics in patients with DRE showed a positive impact on seizure control (16).

Interestingly, a recent study observed that functional gastrointestinal disorders related to microbiota-gut-brain axis dysregulation were significantly associated with the temporal lobe (17). There may be some relationships between microbiota-gut-brain axis dysregulation and neurobehavioral comorbidities, considering the role of the temporal lobe in cognitive function and emotion. However, there is still a lack of relevant research on whether modulating the gut microbiota has a therapeutic effect on epilepsy-associated comorbidities.

Probiotics, regarded as living microorganisms with unknown harmful side effects, provide health benefits to animals and humans by interacting with the intestinal microbiome (18). Modulation of *Bifidobacterium* spp. and *Lactobacillus* spp. were suggested as an effective therapeutic strategy for treatment of DRE (14). BIFICO capsules, containing *Bifidobacterium longum, Lactobacillus acidophilus*, and *Enterococcus faecalis* have been widely used in China for more than 20 years (19). *Enterococcus* spp. increases the colonization of *Bifidobacterium* spp. and provides an anaerobic environment suitable for *Bifidobacterium* spp. *L. acidophilus* produces some growth factors that promote the proliferation of *Bifidobacterium* spp. Combination of these three strains maximizes the prebiotic effect for maintaining gut microbiota balance (20). In this study, we aimed to investigate the effect of BIFICO on cognitive function and emotional symptoms in patients with TLE to provide guidance for treating epilepsy-related comorbidities, thereby improving quality of life.

## Methods

### Participants and inclusion and exclusion criteria

Subjects were recruited from the epilepsy clinic of the neurology department at Capital Medical University Affiliated Beijing Friendship Hospital from January 2020 to December 2021. This was a double-blind, randomized controlled study with an experimental and a placebo control group. This study was approved by the Human Research Ethics Committee of the Capital Medical University Affiliated Beijing Friendship Hospital. All enrolled patients signed an approved informed consent document after meeting the inclusion criteria.

The inclusion criteria were as follows: (1) aged 50–75 years; (2) TLE diagnosis; (3) seizures that occurred at least twice a month for ≥2 years before entering the study; (4) absence of epilepsy induced by other causes, including encephalitis, stroke, brain tumors, diabetes, or metabolic syndrome; (5) absence of generalized motor seizures, mental motor seizures, or other idiopathic syndromes; (6) no history of neurological or psychiatric disorders and; (7) an ability to read and understand the study documents evaluated by the investigators.

The exclusion criteria were as follows: (1) use of topiramate or phenobarbital, which affect cognition; (2) use of other probiotics or yogurts with live or immune-enhancing supplements within the past 3 months; (3) use of antibiotics or anti-inflammatory therapy within the past 3 months.

**Figure 1:**
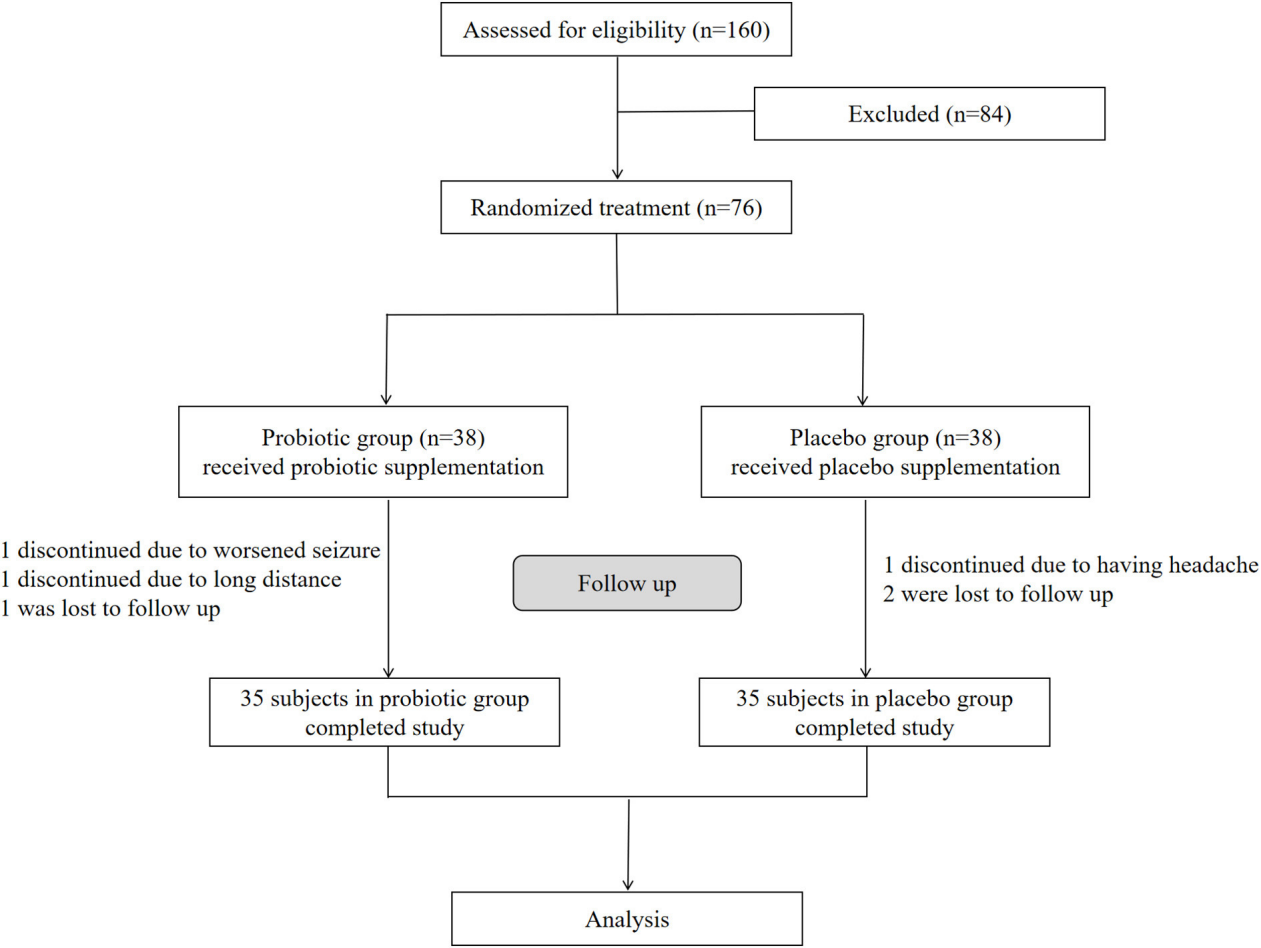
Enrollment, randomization, and follow-up of the participants. Seventy six enrolled participants were equally and randomly assigned to the probiotic and placebo groups. After follow-up, 35 participants in probiotic group and 35 in placebo group finally completed the study respectively for analysis.

We assessed 160 participants for eligibility, and 76 were enrolled in this study according to the inclusion and exclusion criteria (Figure 1). The 76 enrolled participants were equally and randomly assigned to the probiotic and placebo groups. During the 12 weeks of treatment, three participants from the placebo group dropped out of the study; one due to worsened seizures, one due to the long distance involved, and one was lost to follow-up. Additionally, three participants from the probiotic group dropped out in this period; one discontinued due to headaches, and two were lost to follow-up. Therefore, this study eventually included 70 participants (35 enrolled in the placebo group and 35 in the probiotic group (Figure 1).

### Management and procedures

Participants in the probiotic group were assigned to take two capsules after breakfast and dinner per day for 12 weeks (each capsule contained *B. longum, L. acidophilus*, and *E. faecalis*; every living bacterium had >1 × 10^7^ colony-forming units). In the placebo group, each capsule contained only 210 mg of starch. The probiotic products and placebos could not be distinguished by package, color, taste, or smell (provided by Shanghai Xinyi Pharmaceutical Co., Ltd., Shanghai, China). Antiseizure treatment for the participants was not changed during the treatment period for either group.

All participants were evaluated for seizure frequency, cognitive function, anxiety, depression, and quality of life, before and after the intervention. All participants, investigators, and researchers were blinded throughout the study period. After the study was completed, all data were used for statistical analysis.

### Outcome assessments

Demographic information included sex, age, education years, and body mass index (BMI, kg/m^2^). The clinical features included seizure frequency, epileptic history, seizure focus location, and number of AEDs.

The number of episodes in the month before enrollment was regarded as the baseline, and the number of episodes in the third month with probiotic/placebo treatment was regarded as the endpoint. We evaluated the changes of seizure frequency between the baseline and the endpoint.

The Wechsler Adult Intelligence Scale-Fourth Edition (WALS-IV) was used to assess cognitive function (21). WMS-IV contains ten subtests that contribute to four cognitive spheres: the verbal comprehension index (VCI), perceptual reasoning index (PRI), working memory index (WMI), and processing speed index (PSI). Full-scale IQ (FSIQ) was calculated for all subtests. The scores of the four cognitive spheres and the FSIQ were transformed into an index according to the test manual (M = 100, SD = 15).

The Wechsler Memory Scale-Fourth Edition (WMS-IV) was used to assess participants' memory performance in the probiotic and placebo groups (22). This scale consists of five subtests that produce five indices: the auditory memory index (AMI), visual working memory index (VWMI), visual memory index (VMI), immediate memory index (IMI), and delayed memory index (DMI). According to the test manual, the scores for the five indices were transformed into an index (M = 100, SD = 15).

The Hamilton Depression Scale (HAMD) was used to assess depression severity (23, 24). It contains 24 items, and the total score ranges from 0 to 75. The severity ranges based on the total score for depression were as follows: no depression (<8), mild depression (8–20); moderate depression (21–35); and severe depression (>35).

The Hamilton Anxiety Rating Scale (HAMA) was used to evaluate the level of anxiety (25). This scale consists of 14 symptom-defined items, and the score for each item ranges from zero (not present) to four (severe). Based on the total scores, the severity ranges for anxiety were as follows: absence of anxiety (<7), mild anxiety (7–13), definite anxiety (14–20), obvious anxiety (21–29), and severe anxiety (>29).

The Quality of Life in Epilepsy Inventory (QOLIE)-89 item was used to measure the quality of life. The total score of this inventory provides an estimate of overall health-related quality of life.

### Statistical analyses

All data were analyzed using the Statistical Package for Social Sciences (version 21; IBM, Armonk, NY) and GraphPad Prism (version 9; GraphPad Software, San Diego, CA). Shapiro-Wilk test was used to verify the normal distribution of continuous variables. The data of General characteristics and epileptic information were expressed as means and standard deviations and analyzed by using Student's *t*-tests. The categorical data were analyzed by Chi square test and Fisher test. Significant differences were set at a *p*-value <0.05. Within-patient contrasts were analyzed by using Mann-Whitney U test that were adjusted by using the Bonferroni correction (*p* < 0.005 for the cognitive measures and *p* < 0.008 for the neuropsychiatric measures). Correlations between variables were assessed using Spearman test.

## Results

### General characteristics of participants

We assessed 160 participants for eligibility, and 76 were enrolled in this study and randomly assigned to either the probiotic group (*n* = 38) or the placebo group (*n* = 38). Finally, 70 participants completed the study (probiotics, *n* = 35; placebo, *n* = 35), and six subjects dropped out. The patients' demographic and clinical characteristics are shown in Table 1. General characteristics including mean age (probiotics vs. placebo: 60.5 ± 7.0 vs. 60.2 ± 5.8 years), the ratio of females (probiotics vs. placebo: 48.6 vs. 51.4%), years of education (probiotics vs. placebo: 9.3 ± 2.6 vs. 9.1 ± 2.8 years), and BMI (probiotics vs. placebo: 24.9 ± 1.7 vs. 24.6 ± 1.5 kg/m^2^) were matched between the two groups with no significant difference (*p* > 0.05). Epileptic information such as epileptic history (probiotics vs. placebo: 10.5 ± 5.1 vs. 9.7 ± 4.9 years), seizure frequency (probiotics vs. placebo: 5.4 ± 1.4 vs. 5.6 ± 1.5 years), focus location (left, probiotics vs. placebo: 45.7 vs. 51.4%), and the number of AEDs did not significantly differ between the two groups (*p* > 0.05).

**Table 1:** Demographic and clinical characteristics of participants.

**Characteristic**	**Probiotic group (*****n*** = **35)**	**Placebo group (*****n*** = **35)**	***P*** **-value**
Mean age (±SD, years)	60.5 (7.0)	60.2 (5.8)	0.869
Female sex, *n* (%)	17 (48.6%)	18 (51.4%)	0.811
Education (±SD, years)	9.3 (2.6)	9.1 (2.8)	0.696
BMI (±SD, kg/m^2^)	24.9 (1.7)	24.6 (1.5)	0.328
Epileptic history (±SD, years)	10.5 (5.1)	9.7 (4.9)	0.465
Seizure frequency (±SD, seizures/month)	5.4 (1.4)	5.6 (1.5)	0.568
Focus location, *n* (%)			0.632
Left	16 (45.7%)	18 (51.4%)	
Right	19 (54.3%)	17 (48.6%)	
Number of AEDs*, n* (%)			0.874
1	9 (25.7 %)	10 (28.6 %)	
2	14 (40 %)	15 (42.9 %)	
3+	12 (34.3 %)	10 (28.5 %)	

*Continuous variables were analyzed by Student's t-tests, and categorical variables were assessed by chi-square test. No significant differences between groups*.

### Effects of probiotics on seizure frequency in patients with TLE

**Figure 2:**
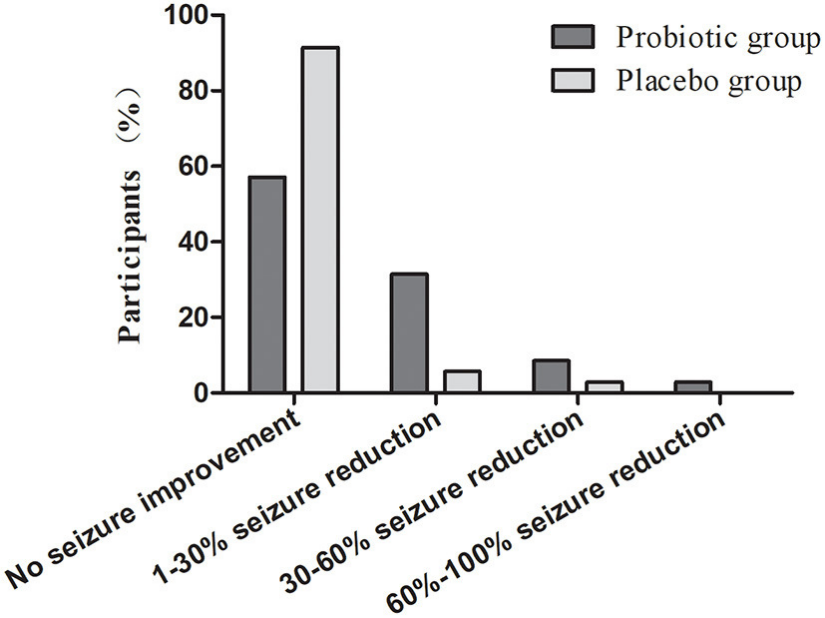
Level of seizure improvement in participants with epilepsy after receiving treatment of probiotic and placebo.

To observe the effect of probiotics on seizure frequency in the participants, the data were classified as no seizure improvement, 1–30% seizure reduction, 30–60% seizure reduction, and 60–100% seizure reduction and analyzed by using chi-square test and Fisher's exact test. As shown in Figure 2, the results were presented as the ratios of participants with no seizure improvement (probiotics vs. placebo: 57.1 vs. 91.4%, *p* < 0.05), 1–30% reduction (probiotics vs. placebo: 31.4 vs. 5.7%, *p* < 0.05), 30–60% seizure reduction (probiotics vs. placebo: 8.57 vs. 2.9%, *p* > 0.05), and 60–100% seizure reduction (probiotics vs. placebo: 2.8 vs. 0%, *p* > 0.05).

### Group differences in the WAIS-IV and WMS-IV index scores

On the WAIS-IV, as shown in Table 2, there were no significant differences between the probiotics and placebo groups in the four cognitive indices and FSIQ at baseline. After 3 months of intervention, the scores in probiotic group on PSI, WMI, and FSIQ were observed higher than those in placebo group, but with no significant differences. Similarity, On the WMS-IV, the participants in the probiotic and placebo groups were at the same level on the five indices at baseline (Table 2). At the endpoint, there was no significant differences between the probiotic and placebo groups on AMI, VWMI, VMI, IMI, and DMI.

**Table 2:** Comparison between groups on WAIS-IV and WMS-IV.

**Cognitive function**	**Baseline**				**Endpoint**			
	**Probiotic**	**Placebo**	**Z**	***P***	**Probiotic**	**Placebo**	**Z**	***P***
WAIS-IV
VCI	91.37 (15.41)	90.97 (15.61)	−0.065	0.948	92.57 (15.03)	92.14 (15.23)	−0.047	0.962
PRI	84.11 (16.25)	85.03 (16.25)	−0.27	0.787	85.46 (15.83)	85.29 (16.22)	−0.024	0.981
PSI	84.60 (15.77)	82.17 (15.85)	−0.565	0.572	93.77 (15.83)	83.29 (15.91)	−2.716	0.007
WMI	84.26 (15.13)	85.80 (16.67)	−0.406	0.685	95.23 (15.48)	86.57 (17.08)	−2.105	0.035
FSIQ	86.09 (10.30)	85.99 (10.71)	−0.012	0.991	91.76 (9.21)	86.82 (10.54)	−1.915	0.049
WMS-IV
AMI	83.03 (16.19)	83.29 (16.07)	−0.194	0.846	92.91 (17.19)	83.57 (16.01)	−2.165	0.03
VMI	87.31 (15.56)	85.20 (18.05)	−0.6	0.549	95.54 (15.36)	87.17 (18.25)	−2.016	0.044
VWMI	82.80 (17.34)	82.94 (17.30)	−0.1	0.92	85.09 (18.04)	83.49 (17.69)	−0.388	0.698
IMI	85.14 (15.14)	84.66 (15.73)	−0.147	0.883	94.11 (16.15)	86.17 (15.20)	−2.454	0.014
DMI	85.11 (15.52)	84.11 (15.05)	−0.076	0.939	94.11 (16.53)	86.14 (15.71)	−2.194	0.028

*The WAIS-IV and WMS-IV scores are presented in corresponding age-adjusted scaled scores (index scores M = 100, SD = 15). “Z” indicates Mann Whitney U test. Bonferroni correction was applied to determine statistical significance*.

### Group differences in the HAMA, HAMD, and QOLIE-89 scales

**Figure 3:**
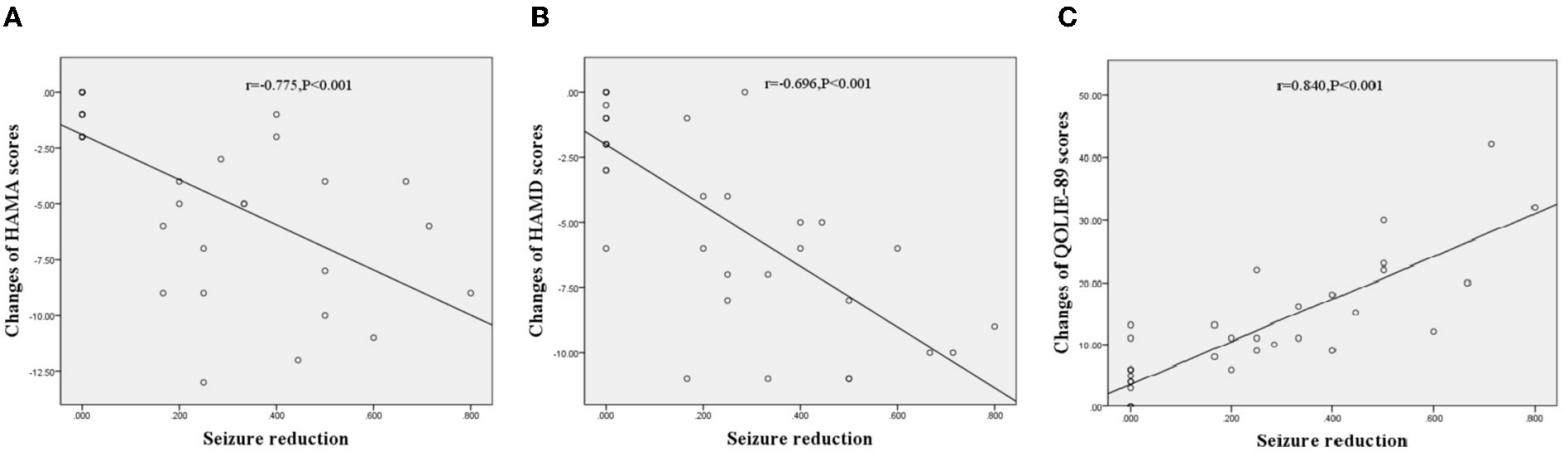
Correlations between changes of neuropsychiatric performances treated with probiotics and seizure reduction in TLE patients. **(A)** Correlations between changes of HAMA scores and seizure reduction, r = −0.775, *p* < 0.001. **(B)** Correlations between changes of HAMD scores and seizure reduction, r = −0.696, *p* < 0.001. **(C)** Correlations between changes of QOLIE scores and seizure reduction, r = 0.840, *p* < 0.001.

Neuropsychological investigations of the participants are shown in Table 3. There were no significant differences between the probiotic and placebo groups in the baseline HAMA, HAMD, and QOLIE-89 scores. After 3 months of intervention, the participants treated with probiotics showed a significant reduction in the HAMA (9.54 ± 5.51 vs. 13.57 ± 6.16, *p* = 0.003) and HAMD (11.83 ± 5.49 vs. 15.23 ± 5.56, *p* = 0.006). There was also an increase in the QOLIE-89 scores (60.29 ± 14.01 vs. 51.91 ± 13.20, *p* = 0.006) compared with those treated with placebo. In addition, an analysis of the effects of seizure reduction on these scales using Spearman test showed that the performance of the participants treated with probiotics had a negative correlation with seizure reduction on HAMA (r = −0.775, *p* < 0.001, Figure 3A) and HAMD (*r* = −0.696, *p* < 0.001, Figure 3B) and a positive correlation with seizure reduction on QOLIE-89 (r = 0.840, *p* < 0.001, Figure 3C).

**Table 3:** Neuropsychological investigation of participants.

**Neuropsychiatric performance**	**Baseline**				**Endpoint**			
	**Probiotic**	**Placebo**	**Z**	***P***	**Probiotic**	**Placebo**	**Z**	***P***
HAMA	13.83 (6.30)	14.37 (6.34)	−0.453	0.65	9.54 (5.51)	13.57 (6.16)	−2.997	0.003*
HAMD	15.97 (6.03)	16.60 (5.94)	−0.778	0.437	11.83 (5.49)	15.23 (5.56)	−2.743	0.006*
QOLIE-89	48.91 (13.37)	49.71 (13.64)	−0.006	0.995	60.29 (14.01)	51.91 (13.20)	−2.758	0.006*

“Z” indicates Mann Whitney U test. Bonferroni correction was applied to determine statistical significance. *p <0.008.

## Discussion

This study was a prospective examination targeting the effect of probiotics on cognitive function and neuropsychiatric manifestations using comprehensive tests in an adjunctive trial in patients with TLE. This study supports the assertion that supplementation with probiotics effectively increase the antiseizure effect and improve intelligence, memory impairment, anxiety, depression, and low quality of life to a certain extent.

The role of the gut-brain axis in epilepsy has been gradually recognized. A study observed elevated α-diversity in the composition of gut microbiota in patients with DRE, especially those with 4 or more seizures per year (14). Linear discriminant analysis showed increases in the relative abundance of Firmicutes and decreases in Bacteroides in patients with DRE (14). Another study detected fecal microbiota in healthy children and in those with DRE, and found differences in fecal microbial β-diversity between groups instead of α-diversity, and also observed increased relative abundance of Firmicutes and Proteobacteria and decreased abundance of Bacteroidetes and Actinobacteria in infants with DRE (26). Modulation of the gut microbiome may be a potential strategy for treating epilepsy. A case study showed that seizures were controlled in a patient diagnosed with Crohn's disease and epilepsy after receiving fecal microbiota transplantation (27). Ketogenic diets have an antiseizure effect in DRE, and the possible mechanism involves changes in microbiota, microbial interactions, and variance in neurotransmitter and neuroactive peptides levels (15, 28, 29). Probiotics has been recognized as another promising therapy in epilepsy. In an open-label study, 28.9% of patients with DRE displayed more than 50% seizure reduction following treatment with a cocktail of *L. plantarum, L. acidophilus, L. helveticus, L. casei, B. lactis, Streptococcus salivarius, and L. brevis* (16).

*Lactobacillus* and *Bifidobacterium* spp. were found to have positive impacts on neurological disorders and psychological diseases (18, 30, 31). A potential therapeutic strategy for epilepsy could be given by modulating levels of *Lactobacillus* and *Bifidobacterium* spp. (14). BIFICO is a cocktail probiotic capsule which consists of *B. longum, L. acidophilus*, and *E. faecalis*. In this study, we observed that supplementation with BIFICO improved seizure frequency in patients with TLE. Current studies have indicated that gut microbiota-derived metabolites and cellular components maintain brain homeostasis. Gut dysbiosis caused by any insult results in disturbance of metabolites and neurotransmitters involved in neural regulation, including 5-hydroxytryptamine, tryptophan, glutamine, gamma-aminobutyric acid (GABA), histamine, short-chain fatty acids, lipopolysaccharides, branched-chain amino acid, bile acids, and catecholamines (13). Abnormalities in these molecules further affect the function of glia, synaptic pruning, myelination, and the blood–brain barrier, which are closely related to seizure susceptibility (10, 13). Supplementation with probiotics may therefore restore brain homeostasis through balancing gut microbiota.

Cognitive impairment is one of several comorbidities with epilepsy. Gut dysbiosis has been found to be involved in cognitive impairment diseases. Bialecka et al. (32) showed that variance in Bacteroidetes and Firmicutes was correlated with mild cognitive impairment, dementia, and Alzheimer's disease (AD), suggesting that alterations in these phyla leading to gut dysbiosis may contribute to cognitive impairment. Probiotics might be an adjustable intervention for cognitive impairment. Razaeiasi et al. (33) observed that treatment with probiotics (*L. acidophilus, B. bifidum*, and *B. longum*) significantly improved spatial learning and memory in rats with AD. Recently, an animal study showed that bacteriotherapy attenuated seizure activity and partially improved spatial learning and memory in pentylenetetrazole-induced kindled rats (34). This may contribute to the improvement in the antioxidant/oxidant ratio and the increased level of GABA stimulated by beneficial bacterial strains in the gut microbiota (34). However, in this human study, supplementation with probiotics was not observed to significantly improve cognitive function. Maybe some individual participants showed obvious improvement on intelligence or memory, but there was no significantly statistical difference. It is necessary to expand the sample size for further analysis. In addition, in animal experiments, timely intervention may effectively prevent the occurrence of cognitive dysfunction in epilepsy, this may be due to the pathology changes in temporal lobe affecting cognition have not yet formed. However, in clinic, pathological changes related to cognitive deficit may have been existed in patients with TLE. At this stage, it is difficult to reverse the pathological changes and therefore may not improve cognitive deficit. More experiments are needed for verification in the future.

Patients with epilepsy have a higher prevalence of anxiety and depression (35). Previous studies have shown that anxiety and depression are risk factors for refractory epilepsy and have suggested a pathological association between neuropsychiatric comorbidities and uncontrolled seizures (36). This special pathological link highlights great challenges in the intervention of epilepsy-associated depression and anxiety. Recently, gut microbiota was found to be involved in the development of neuropsychiatric disorders (31, 37). *Lactobacillus* and *Bifidobacterium* spp. are regarded as psychobiotics with positive impacts in patients with depression and anxiety (38, 39). In this study, we observed that measurements of anxiety, depression, and low quality of life in patients with TLE were significantly improved by supplementation with BIFICO probiotics. These changes were closely related to seizure control. We speculate that there are two possible reasons for this: first, the improvement in anxiety and depression may only contribute to seizure reduction, and second, probiotic-modulated changes in metabolism and neurotransmitters such as dopamine, serotonin, noradrenaline, and GABA may have an effect on TLE pathology (40, 41).

This study had some limitations. First, the sample size was small, and future studies are needed to expand the sample size to verify our results. Second, we conducted our research based on previous studies and did not analyze the metabolic and neurotransmitter effects of probiotics on epilepsy-associated comorbidities. In the future, more studies are needed to study the potential mechanism of the positive impact of probiotics and to build a pathological link between the gut microbiome, epilepsy, and epilepsy-associated comorbidities. Third, clinical seizures were used in this study as the observation index to evaluate whether the improvement of cognitive function and neuropsychological disorders in patients with TLE treated with probiotics were affected by seizure reduction. The impact of interictal activity was not observed in this study, which needs future studies to evaluate and discuss.

### Conclusions

In conclusion, the results of this study suggested that BIFICO probiotics have a positive effect on seizure control, anxiety, depression, and quality of life in patients with TLE. This study indicated that BIFICO probiotics was beneficial to TLE patients as adjuvant therapy.

## Data availability statement

The original contributions presented in the study are included in the article/supplementary material, further inquiries can be directed to the corresponding author/s.

## Ethics statement

The studies involving human participants were reviewed and approved by Human Research Ethics Committee of the Capital Medical University Affiliated Beijing Friendship Hospital. The patients/participants provided their written informed consent to participate in this study.

## Author contributions

XW wrote the draft of this article. RM and XL collected and analyzed the data. YZ revised the manuscript and gave the final approval. All authors contributed to the article and approved the submitted version.

## Conflict of interest

The authors declare that the research was conducted in the absence of any commercial or financial relationships that could be construed as a potential conflict of interest.

## Publisher's note

All claims expressed in this article are solely those of the authors and do not necessarily represent those of their affiliated organizations, or those of the publisher, the editors and the reviewers. Any product that may be evaluated in this article, or claim that may be made by its manufacturer, is not guaranteed or endorsed by the publisher.
